# The Prevalence of Vitamin A Deficiency in Chinese Children: A Systematic Review and Bayesian Meta-Analysis

**DOI:** 10.3390/nu9121285

**Published:** 2017-11-25

**Authors:** Peige Song, Jiawen Wang, Wei Wei, Xinlei Chang, Manli Wang, Lin An

**Affiliations:** 1 Department of Maternal and Child Health, School of Public Health, Peking University, Beijing 100191, China; peigesong@hsc.pku.edu.cn (P.S.); changxl@bjmu.edu.cn (X.C.); wangmanli16@163.com (M.W.); 2 Centre for Population Health Sciences, University of Edinburgh, Edinburgh EH8 9AG, UK; 3 Institute of Medical Humanities, Peking University, Beijing 100191, China; beatrice.wang@pku.edu.cn; 4 School of Foundational Education, Peking University, Beijing 100191, China; weiweichina@pku.edu.cn

**Keywords:** Vitamin A deficiency, children, China

## Abstract

Vitamin A deficiency (VAD), a leading cause of preventable childhood blindness, has been recognized as an important public health problem in many developing countries. In this study, we conducted a systematic review to identify all population-based studies of VAD and marginal VAD (MVAD) in Chinese children published from 1990 onwards. Hierarchical Bayesian meta-regressions were performed to examine the effects of age, sex, setting and year on the prevalence of VAD and MVAD, separately. The estimated prevalence was applied to the Chinese pediatric population in the year 2015 to generate prevalence estimates of VAD and MVAD for defined age groups, with 95% credible intervals (CrIs). Fifty-four studies met the inclusion criteria. The prevalence of VAD and MVAD both decreased with increasing age, and rural children had a higher prevalence of VAD and MVAD than urban children. In 2015, the prevalence of VAD was 5.16% (95% CrI: 1.95–12.64) and that of MVAD was 24.29% (95% CrI: 12.69–41.27) in Chinese children aged 12 years and under. VAD remains a public health problem in China. Efforts to reduce VAD in younger children are needed, especially for those in rural areas.

## 1. Introduction

Vitamin A, i.e., retinol and its derivatives, is a key nutrient for the maintenance of immune system, normal vision, growth and survival in humans [1,2,3]. Since vitamin A cannot be synthesized by the body, people of all ages can be affected by the deficiency of vitamin A [2,4,5]. Chronic low intake of vitamin A from diets is the main underlying cause of vitamin A deficiency (VAD), especially in nutritionally demanding periods of life, such as early childhood and pregnancy [6,7,8,9]. VAD is linked to xerophthalmia, night blindness, impaired immune functions and anemia, and increases child mortality from measles and diarrhea [7,10,11,12].

According to the World Health Organization (WHO) estimates, 190 million (33.3%) preschool-age children are vitamin A deficient [8]. This estimate was based on VAD prevalence estimates in countries with a gross domestic product (GDP) of less than US $15,000 in 2005. Globally, VAD has been recognized as a leading cause of preventable childhood blindness, and remains an important public health problem in many developing countries [8,13,14]. For the largest developing country, China, it is estimated that VAD, defined as a serum retinol concentration of less than 0.70 μmol/L, affects fewer than 10% of preschool-aged children, which marks VAD being of mild public health significance in China according to the WHO classification [8,15].

However, for a large developing country such as China, where economic growth is fundamentally uneven and disparities between urban and rural areas are marked, one single estimate of VAD prevalence at the national level is not sufficient [16,17]. To address the situation of VAD prevalence comprehensively, prevalence estimates of VAD in subnational populations are therefore called for [6]. The past decades have seen a growing body of epidemiological studies on VAD prevalence in China [18,19,20]. These individual studies, however, are generally limited to the characteristics of their study populations. 

To formulate public health policies and interventions, we conducted a systematic review of existing literature in both English and Chinese bibliographic databases. The prevalence of VAD in Chinese children was estimated by a Hierarchical Bayesian (HB) approach, and the variations of VAD prevalence by age, gender and setting were also assessed. Following the same approach, the prevalence of marginal VAD (MVAD, a low vitamin A concentration status), defined as a serum retinol concentration of between 0.70 μmol/L and 1.05 μmol/L, was also estimated to signal children who might have inadequate vitamin A intake.

## 2. Materials and Methods

### 2.1. Literature Search and Study Selection

This systematic review was conducted following the Preferred Reporting Items for Systematic Reviews and Meta-Analyses (PRISMA) guidelines [21]. The search sources included three Chinese bibliographic databases (China National Knowledge Infrastructure (CNKI), Wanfang data and Chinese Biomedicine Literature Database (CBM-SinoMed)) and three English databases (PubMed, Medline and Embase). Articles on the prevalence of VAD in Chinese children were retrieved by using the combination of terms for vitamin A (e.g., vitamin A, retinol and aquasol A), China (e.g., China and Chinese), children (e.g., children, infants and toddlers) and prevalence (e.g., prevalence, rate and epidemiology). Different search strategies were developed for each bibliographic database to suit their specific searching characteristics (see Table S1 for full details). The reference lists of included articles were also reviewed for identifying articles that were missed in the database searches. Only articles that were published from 1990 onward were retained for further assessment of eligibility. No language restrictions were imposed.

Articles titles, abstracts, and full-text were independently screened by two researchers (J.W. and W.W.), and any discrepancies were resolved by consensus. We included all population-based investigations of VAD epidemiology in children residing in Mainland China, from which the numerical estimates of VAD/MVAD prevalence could be obtained. In this study, the cut-offs of serum retinol concentrations for defining VAD (serum retinol < 0.70 μmol/L) and MVAD (serum retinol within 0.70–1.05 μmol/L) were in line with the recommendation of WHO [8]. Therefore, studies that did not adopt the WHO cut-offs of serum retinol concentrations for VAD and MVAD, and those with no clear assessment methods of biomedical vitamin A levels were excluded; likewise, studies that relied on self-reported diagnoses of VAD or MVAD were also excluded. Studies that were conducted in a sub-group with characteristics that clearly indicated it to be unrepresentative of the general pediatric population were excluded. For multiple publications of the same investigation, the one with the most comprehensive data was kept.

### 2.2. Data Extraction

Extraction of data was conducted independently by the same two researchers (J.W. and W.W.). A structured data extraction form was developed and piloted to gather relevant data from each article. Data abstracted contained the basic information regarding the study (e.g., title, author, publication year, investigation site, investigation year, study design, and sampling method), the investigated children (age, sex and setting (urban, rural and mixed)) and the reported prevalence estimates of VAD/MVAD. Wherever available, stratified prevalence by age group, sex and setting was extracted. For studies that did not specify the investigation year, the year of investigation was generated by subtracting three years from the year of publication. This was done based on the average difference of publication year and investigation year from studies where data were reported. Discrepancies were discussed by consensus.

### 2.3. Data Analyses

Due to the hierarchical structure of extracted data, we used HB approaches to pool the prevalence of VAD and the prevalence of MVAD in Chinese children, separately. In the Bayesian meta-analysis, variations at all levels can be simultaneously incorporated, therefore heterogeneity can be dealt with more adequately [22]. Briefly, the number of children with VAD/MVAD (r*_ij_*) was specified as binomially distributed:

*r_ij_* ~ binomial(*n_ij_*, *p_ij_*) (1)

where *n_ij_* was the total number of investigated children and *p_ij_* was the corresponding prevalence of VAD/MVAD in the *i*th study for the *j*th category of the variable of interest (age, sex, setting and investigation year category). The logit of *p_ij_* therefore follows a normal distribution. Non-informative prior was specified for all the parameters.

To assess the effects of age, sex and setting, these variables of interest were subsequently added to the abovementioned base model. Deviance information criterion (DIC) was adopted to compare the goodness of models, with a small DIC representing a better model fit. As a rule, a difference of DIC between two models of larger than 10 indicates a definite rule out of the model with higher DIC [23]. Although preschool-age children are at a higher risk of VAD, the evaluation of VAD in school-aged children also deserves attention [4,24]. In this study, we extended the age range of our targeted children to 12 years old and under. The secular trends in VAD/MVAD prevalence were also examined by taking the investigation year into the base model. Finally, the estimated setting- and age-specific prevalence and 95% credible intervals (CrIs) were applied to the Chinese pediatric population in the year 2015 [25], to generate prevalence estimates of VAD and MVAD for defined age groups.

All HB models were fitted with Markov chain Monte Carlo simulations, the median of the posterior distributions of the estimates of interest along with 95% CrI was reported. Inferences were based on 70,000 iterations, the first 20,000 of which were used as burn-in. HB analyses were performed using the JAGS software (version 4.2.0, http://mcmc-jags.sourceforge.net/) from R (version 3.3.0, R Foundation for Statistical Computing, Vienna, Austria).

## 3. Results

### 3.1. Eligible Studies and Characteristics

**Figure 1:**
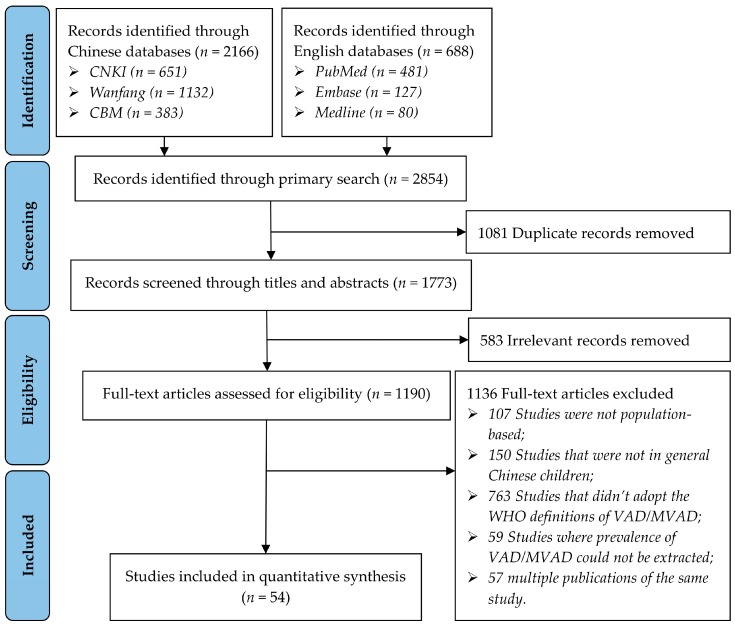
PRISMA flow diagram of study selection process. PRISMA, Preferred Reporting Items for Systematic Reviews and Meta-Analyses; CNKI, China National Knowledge Infrastructure; CBM, Chinese Biomedicine Literature Database; WHO, World Health Organization; VAD, vitamin A deficiency; MVAD, marginal vitamin A deficiency.

The literature search identified a total of 2854 records for screening (Figure 1). After duplicates were removed, 1773 unique records were screened by titles and abstracts. After exclusions of obviously irrelevant records, a total of 1190 records were assessed for eligibility in full text. Finally, 54 articles met the inclusion criteria and were included for further analysis (see Table S2 for the full list).

The main characteristics of every study are outlined in Table S3. Briefly, the 54 studies included in this review were all cross-sectional in design, published between 1995 and 2016, reported data from 1992 to 2015 across the nation, and included a total of 151,055 participants. The prevalence of VAD was reported in all of the included studies, of which 45 reported the prevalence of MVAD.

### 3.2. The Effects of Age and Setting on the Prevalence of VAD and MVAD

Based on informative data points from the 54 studies, the effects of age, gender, setting and investigation year on the prevalence of VAD were assessed using the HB meta-regression, separately. The comparisons of model fit suggested that age and setting were significantly associated with the prevalence of VAD (Table S4). The best fit model for predicting the prevalence of VAD was generated:

Logit(*p*) = (−0.239) × Age − 0.100 × Setting*_urban_*+ 0.564 × Setting*_rural_* − 1.027 (2)

where Logit(*p*) = ln(*p*/(1 − *p*)), *p* is the prevalence of VAD; Setting*_urban_* = 1 for urban areas and 0 otherwise; and Setting*_rural_* = 1 for rural areas and 0 otherwise.

For MVAD, the effects of age, gender, setting and investigation year on the prevalence estimates were assessed based on the 45 studies that provided relevant information. Similarly, the HB meta-regressions indicated that age and setting had significant effects on the prevalence of MVAD (Table S4). The best fit model for predicting the prevalence of MVAD was:

Logit(*p*) = (−0.105) × Age − 0.535 × Setting*_urban_* + 0.016 × Setting*_rural_* − 0.559 (3)

where Logit(*p*) = ln(*p*/(1 − *p*)), *p* is the prevalence of MVAD; Setting*_urban_* = 1 for urban areas and 0 otherwise; and Setting*_rural_* = 1 for rural areas and 0 otherwise.

**Figure 2:**
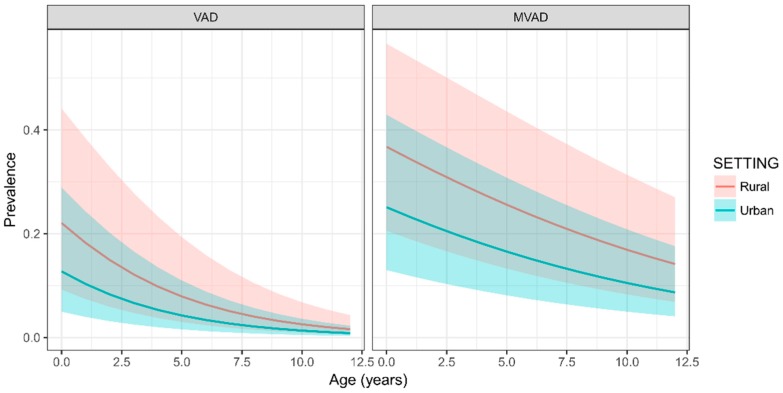
The setting-specific relationship between age and prevalence of VAD and MVAD in Chinese children.

Based on the best fit models for predicting the prevalence of VAD and MVAD, the setting-specific relationships between age and the prevalence of VAD and MVAD are shown in Figure 2. Generally, the prevalence estimates of VAD and MVAD both decreased with increasing age, and there was a marked difference of prevalence estimates between urban and rural children, where children in rural areas had higher VAD and MVAD prevalence than those in urban areas.

### 3.3. The Prevalence of VAD and MVAD in Chinese Children in 2015 and the Public Health Significance

**Figure 3:**
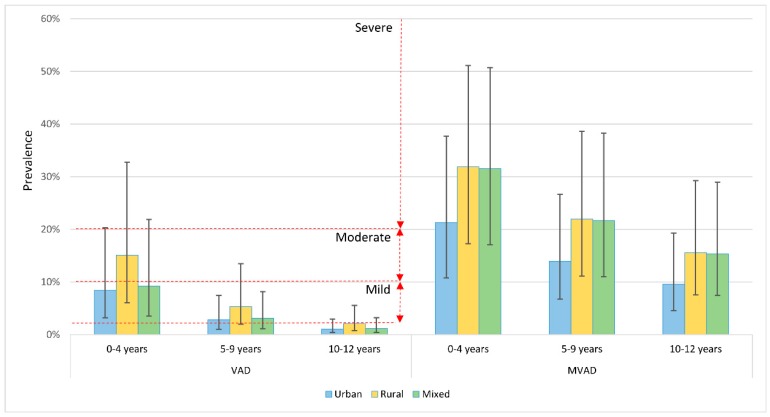
Estimated setting-specific prevalence of VAD and MVAD in Chinese children in 2015, by age group.

After applying the estimates of setting- and age-specific VAD and MVAD prevalence on Chinese pediatric population in the year 2015, the prevalence of VAD and MVAD in children aged 12 years and under was calculated (Table 1 and Figure 3). In 2015, the prevalence of VAD was 5.16% (95% CrI: 1.95–12.64) and that of MVAD was 24.29% (95% CrI: 12.69–41.27) in Chinese children aged 12 years and under. The prevalence of VAD ranged from 9.23% (95% CrI: 3.54–21.88) in children under five years to 1.18% (95% CrI: 0.43–3.23) in those aged 10–12 years. The VAD prevalence between urban and rural areas differed consistently across the whole age range, where the prevalence of VAD in rural children was around two-fold higher than that in urban children. In the case of MVAD, the prevalence estimates also declined with increasing age, from 31.53% (95% CrI: 17.06–50.73) in under-five children to 15.36% (95% CrI: 7.48–28.94) in children aged 10–12 years. Similarly, the prevalence of MVAD was higher in rural areas than in urban areas.

Based on the WHO cut-offs of defining VAD public health significance (Figure 3), China can be classified as having a mild public health problem in terms of biochemical VAD in preschool-age children, while the prevalence of VAD for rural preschool-age children is of moderate public health significance (≥10%–<20%) [8].

## 4. Discussion

Based on an extensive meta-analysis, this study presents comprehensive estimates of the prevalence of VAD and MVAD in Chinese children. Overall, the prevalence of VAD was 5.16% and that of MVAD was 24.29% in children aged 12 years and under. The prevalence of VAD and MVAD both decreased with increasing age. Higher prevalence estimates of VAD and MVAD were observed in rural children than in urban children. According to the results presented herein, VAD is of mild public health significance in China. However, the planning and organization of preventative strategies in rural settings are still needed.

To the best of our knowledge, this study provides the most up-to-date national representative estimates of VAD and MVAD prevalence in China. By pooling data based on many participants from diverse studies conducted across the whole nation, this study included the most comprehensive information than any individual investigations. Although a high level of heterogeneity existed among the included studies, the HB meta-analytic approach quantified and accounted the effects of different study characteristics (age, gender, setting and secular trend) simultaneously. Therefore, the estimates generated are a precise reflection of the national prevalence of VAD and MVAD. 

The WHO Global Database on VAD developed a regression equation to predict the prevalence of VAD in the population at risk. For preschool-age children, the prediction equation of VAD prevalence is dependent on GDP, under five mortality and population growth rate in a specific country [8]. By taking the latest GDP, under five mortality and population growth rate in China in 2015, it is estimated that 9.36% of the Chinese preschool-age children were with VAD. This WHO estimate coincides with the 9.23% reported in our analysis.

This HB meta-analysis confirms that the prevalence of VAD and MVAD is highly associated with age and is most common in young children. These findings lend support to the notion that the general nutritional status and vitamin A status naturally improves with increasing age [26,27]. One main reason for this phenomenon is the transition from breastfeeding to dependence on other dietary sources of the vitamin, so children can start to satisfy their vitamin A needs by consuming sufficient dietary sources when they grow older [5]. 

According to the WHO standard, VAD in preschool-age children is a mild public health problem in China, which is in accordance with previous global estimates [8,15]. However, this does not imply that the goal of overcoming VAD has been perfectly achieved in China. In this study, substantial inequities between urban and rural settings were revealed regarding the issue of VAD. This can be seen as a reflection of socioeconomic gaps between urban and rural areas, where the availability of vitamin A through diets might be limited in relatively poor rural settings. Community education on the awareness of VAD should be practically conducted in rural areas, so that the caregivers can timely detect signs of VAD at its early stages [27]. Furthermore, localized strategies for preparing micronutrient-rich foods and access to vitamin A supplementation are also integral parts in reducing the gravity of VAD in those settings [4,5,27]. However, these comprehensive nutritional approaches should be targeted and implemented with cautions, because regular intake of high-dose vitamin A supplements is not recommended for all children [9,28,29].

Although growth rates for boys are generally higher than those in girls before the age of 10 years, no sex differential in developing VAD has been confirmed in previous studies [5,30]. The results of our studies are in line with this statement, where no association between sex and prevalence of VAD and MVAD was revealed. Likewise, no appreciable secular trend in prevalence of VAD and MVAD has been detected in our study. However, this finding is not entirely consistent with the global declining trend of VAD prevalence during the past two decades [15]. More individual studies should be enrolled to reveal the time trend of VAD and MVAD prevalence in China if needed.

Limitations should be considered when interpreting the findings. First, the included studies were different in study design and involved different groups of children (e.g., from sites with different economic development levels, from areas with different dietary habits). Although we made a great effort to explore the effects of different characteristics, many unexamined individual-level factors, such as children’s health conditions (e.g., intestinal infestations or infections) and vitamin supplementary intake, may also contribute to the heterogeneity of VAD/MVAD prevalence among studies [31]. Furthermore, the regression-based equations are appropriate for our national estimates of VAD and MVAD prevalence in children by age group and setting, but may not be able to accurately generate estimates of VAD prevalence at local sites because of the restricted availability of primary data in the included studies. More epidemiological studies are still required, especially in the poor and remote areas where VAD may still be a significant public health problem, and therefore can benefit from large-scale targeted supplementation or fortification strategies [4,10,27,32].

## 5. Conclusions

In conclusion, the results of our systematic review and HB meta-analysis show that VAD remains a public health problem in China. Efforts to reduce VAD in younger children residing in rural areas are particularly needed.

## Figures and Tables

**Table 1:** Estimated setting-specific prevalence of VAD and MVAD in Chinese children in 2015, by age group.

Age Range	Prevalence of VAD (%, 95% CrI)			Prevalence of MVAD (%, 95% CrI)		
	Urban	Rural	Mixed	Urban	Rural	Mixed
0–4 years	8.45	15.10	9.23	21.28	31.86	31.53
	(3.22–20.29)	(6.05–32.75)	(3.54–21.88)	(10.77–37.69)	(17.28–51.11)	(17.06–50.73)
5–9 years	2.83	5.33	3.11	13.98	21.94	21.68
	(1.03–7.49)	(1.98–13.48)	(1.14–8.18)	(6.76–26.68)	(11.15–38.61)	(11.00–38.25)
10–12 years	1.08	2.06	1.18	9.62	15.56	15.36
	(0.39–2.94)	(0.75–5.55)	(0.43–3.23)	(4.53–19.27)	(7.59–29.25)	(7.48–28.94)
Overall (0–12 years)	4.99	8.11	5.16	16.36	24.07	24.29
	(1.88–12.30)	(3.17–18.62)	(1.95–12.64)	(8.10–30.10)	(12.55–40.99)	(12.69–41.27)

## Supplementary Materials

The following are available online at http://www.mdpi.com/2072-6643/9/12/1285/s1, Table S1: Search strategy to identify studies reporting the prevalence of childhood VAD in China, Table S2: Full list of the included studies (*n* = 54), Table S3: Detailed characteristics of the included studies (*n* = 54), Table S4: The deviance information criterion (DIC) difference relative to the intercept-only model.
