# Supplementary material for: The Prevalence of Vitamin A Deficiency in Chinese Children: A Systematic Review and Bayesian Meta-Analysis
Source: Nutrients. 2017 Nov 25;9(12):1285. doi: 10.3390/nu9121285 (PMC5748736; doi:10.3390/nu9121285)
Supplement: Supplementary file 1 [file nutrients-09-01285-s001.pdf]

Supplementary Document

Song et al. The Prevalence of Vitamin A Deficiency in Chinese Children: A Systematic Review and Bayesian Meta-analysis

Table S1. Search strategy to identify studies reporting the prevalence of childhood VAD in China.

**CNKI**<br>Access Date: 27 Mar 2017<br>Subject category: Medicine & Public Health<br>Sub-database: Journal, Featured journal, Doctoral dissertation, Master dissertation<br>Search strategy:<br>(SU % '维生素 A ' + '视黄醇') AND (SU % '儿童' + '学生'+ '少年'+ '青少年'-'大学生') AND (SU % '患病率'+ '罹患率' + '现患率'+ '流行' + '调查'+ '现况')<br>Publication date: 1990-01-01 to 2017-03-27<br><br>**Wanfang**<br>Access Date: 28 Mar 2017<br>Sub-database: Journal articles, Dissertations<br>Search strategy:<br>(主题:(维生素 A) + 主题:(视黄醇)) * (主题:(儿童) + 主题:(学生) + 主题:(少年) +主题:(青少年) ^主题:(大学生))* (主题:(患病率) +主题:(罹患率) + 主题:(现患率) + 主题:(流行) + 主题:(调查)+ 主题:(现况))<br>Publication date: 1990-2017<br><br>**CBM-SinoMed**<br>Access Date: 28 Mar 2017<br>Journal category: All journals<br>Search strategy:<br>(维生素 A or 视黄醇) AND (儿童 or 学生 or 少年 or 青少年 not 大学生) AND (患病率 or 罹患率 or 现患率 or 流行 or 调查 or 现况)<br>Publication date: 1990-2017<br><br>**PubMed**<br>Access Date: 27 Mar 2017<br>Search Terms:<br>((vitamin a OR retinol OR retinal OR aquasil a) AND (china OR chinese) AND (child OR children OR adolescen*) AND (prevalen* OR rate* OR epidemiolog*)) AND ("1990/01/01"[PDAT] : "2017/03/27"[PDAT])<br><br>**Embase (Ovid)**<br>Access Date: 27 Mar 2017

#	Searches
**1**	exp retinol/ or vitamin a.mp.
**2**	Chin*.mp.
**3**	exp China/
**4**	exp Chinese/
**5**	child*.mp.
**6**	child/
**7**	adolescen*.mp.
**8**	adolescent/
**9**	exp prevalence/ or prevalen*.mp.
**10**	rate*.mp.
**11**	exp epidemiology/ or epidemiolog*.mp.
**12**	2 or 3 or 4
**13**	5 or 6 or 7 or 8
**14**	9 or 10 or 11
**15**	1 and 12 and 13 and 14
**16**	limit 15 to yr="1990 -Current"

Medline (Ovid)
Access Date: 27 Mar 2017
Search Terms:

#	Searches
**1**	exp Vitamin A/ or vitamin a.mp. or exp Vitamin A Deficiency/
**2**	Chin*.mp.
**3**	exp China/
**4**	exp Child/ or child*.mp.
**5**	exp Adolescent/ or adolesc*.mp.
**6**	exp Prevalence/ or prevalen*.mp.
**7**	rate*.mp.
**8**	exp Epidemiology/ or epidemiolog*.mp.
**9**	2 or 3

**10**	4 or 5
**11**	6 or 7 or 8
**12**	1 and 9 and 10 and 11
**13**	limit 12 to yr="1990 -Current"

Table S2. The full list of included studies (n=54).

Study ID	Author	Year Published	Title
V01	Liu YF et al.	2012	Evaluation on the physical development and nutritional status of 350 preschool children (350 名学龄前儿童体格生长和营养水平现状调查)
V02	Yang C et al.	2016	Comparison on the status of vitamin A in 6- to 13- year-old children between 2002 and 2012 in China (Comparison on the status of vitamin A in 6- to 13- year-old children between 2002 and 2012 in China)
V03	Zhang YW	2003	A Study on the Nutritional Status of Vitamin A and Related Factors among Children Aged 0 to 5 Years in Anhui Province (安徽省 0~5 岁儿童维生素 A 营养状况及其影响因素研究)
V04	Xu JH et al.	2003	Epidemiologic investigation on subclinical vitamin A deficiency among kindergarten children of Anshan city* (鞍山市幼儿园儿童亚临床维生素 A 缺乏症的流行病学调查)
V05	Sun LF et al.	2016	Serum vitamin A level in children aged 2-6 years old in Pinggu District of Beijing (北京市平谷区 2~6 岁儿童血清维生素 A 水平分析)
V06	Chen Z et al.	2002	Investigation on Vitamin A Deficiency and Its Related Factors Among Children* (儿童维生素 A 缺乏及相关因素的调查研究)
V07	Qiu XG et al.	2010	The second investigation on vitamin A deficiency among children under five years old in Fujian (福建省第 2 次 5 岁以下儿童维生素 A 缺乏症调查)
V08	You Y	2010	An Analysis Investigation of the Level of Vitamin A of School-age children between Dongxiang and Bonan, Gansu (甘肃东乡族和保安族农村学龄儿童 VitA 水平分析)
V09	Liu T et al.	2016	Analysis of Serum Vitamin A Concentrations of School-Aged Children in Gansu Rural Area (甘肃省某农村地区学龄儿童维生素 A 营养状况调查)
V10	Qi ZQ et al.	2001	Investigation on the status of vitamin A among children under 5 years in plateau area* (高原地区 5 岁以下儿童维生素 A 水平现状的调查研究)
V11	Fang ZF et al.	2007	Investigation on the relationship between vitamin A and iron deficiency among rural children of Guangxi* (广西农村儿童维生素 A 和铁缺乏营养状况关系的调查研究)
V12	Fu SL et al.	2006	Prevalence of Vitamin Deficiency and the Influencing Factors Among Children Aged 0-5 Years in Hefei (合肥市 0~5 岁儿童维生素 A 缺乏情况及影响因素分析)

V13	Yang Q et al.	2002	Investigation on the nutritional status of vitamin A among children aged 0-6 years in Hubei province* (湖北省 0~6 岁儿童维生素 A 营养状况调查)
V14	Lu L et al.	2001	Investigation on vitamin A deficiency among urban children aged 0-4 years in Huaiyin city* (淮阴市城区 0~4 岁儿童维生素 A 缺乏症情况的调查)
V15	Hu HF	2002	Investigation of vitamin A deficiency and its correlative factors in children of Linyi city (临沂市儿童维生素 A 缺乏及其影响因素的研究)
V16	He YF et al.	2005	Vitamin A Deficiency and Its Influencing Factors Among Children in Nantong (南通市儿童维生素 A 缺乏现状及其影响因素分析)
V17	Cai LR et al.	2010	Epidemiologic investigation on vitamin A deficiency in children under 5 years old in Quanzhou city (泉州市 5 岁以下儿童维生素 A 缺乏流行病学调查)
V18	Huang HY et al.	2004	An Analysis Investigation on the Nutritional Status of Vitamin A among Children Aged 0-5 Years in Xiamen* (厦门市 0~5 岁儿童维生素 A 营养状况调查分析)
V19	Fan P et al.	2012	Serum retinol and carotenoid of rural infants and young children in Linyi of Shandong Province and analysis on their related influencing dietary factors (山东临沂市 254 名农村婴幼儿血清视黄醇和类胡萝卜素水平及其相关膳食因素的关系)
V20	Zhou YX et al.	2002	Investigation on the status of serum vitamin A among partial children aged 0-5 years of Shandong province* (山东省城乡部分 0~5 岁儿童血清维生素 A 水平调查)
V21	Zhao JJ et al.	2016	The status of vitamin A and D among rural students of Shanxi* (陕西农村学生维生素 A 与维生素 D 水平现状)
V22	Zhang YF et al.	2002	An Analysis Investigation on Vitamin A deficiency among Children in three cities of Sichuan* (四川三市县儿童维生素 A 缺乏调查及现状分析)
V23	Zhou ZX	2001	Research on epidemiology of vitamin A deficiency among children aged 0-5 years in Wuhan city (武汉市 0-5 岁儿童维生素 A 缺乏的流行病学研究)
V24	Yang SP et al.	2011	Study on the vitamin A deficiency and its influencing factors among children in Wuhan (武汉市 5 岁以下儿童维生素 A 营养状况及其影响因素研究)
V25	Mi J et al.	2003	Prevalence of vitamin A deficiency in children under six years of age in Tibet, China (西藏自治区六岁以下儿童维生素 A 缺乏情况调查)

V26	Zhong CM	2010	Relationship Between the Nutritional Status of Vitamin A，Trace Elements and the Level of Anti-HBs in Infants (婴幼儿维生素 A 和微量元素营养状况与乙肝表面抗体水平的关系研究)
V27	Jiang HP et al.	2012	Survey on growth level-anaemia and vitamin A deficiency among 356 primary and middle school students in Wuxi county of Chongqing city (重庆市巫溪县中小学生贫血、维生素 A 营养状况及生长水平调查)
V28	Wu ZY	2012	Analysis of serum Vitamin A levels and its influencing factors in children aged 0-5 in Xinxiang City* (新乡市 0 ~ 5 岁儿童维生素 A 水平及其影响因素分析)
V29	Zhao ZY et al	1998	Comparative study of the value of serum Vitamin A, ferritin and haemoglobin in rural children* (农村儿童血清维生素 A 铁蛋白和血红蛋白值的比较研究)
V30	Liu JW et al	2008	Evaluation of nutritional status of Vitamin A in children aged 1-12 in Hunan Province* (湖南省 1~12 岁儿童维生素 A 营养状况评价)
V31	Wang HD et al	2001	Survey on Vitamin A deficiency and anaemia of children in Ruyang County, Henan Province* (河南省汝阳县儿童维生素 A 缺乏及贫血情况调查)
V32	Chen K et al	2008	Evaluation on nutritional status and physical development of preschool children in suburb of Chongqing city (重庆市近郊学龄前儿童体格生长及营养状况调查)
V33	Chen HL et al	2000	Serum Vitamin A among children aged 0-6 in HaiDao, Zhoushan City* (舟山市海岛 0-6 岁儿童血清维生素 A 检测)
V34	Wang R et al	2010	Serum vitamin A among young children aged seven to twelve in China (中国裕固族 7-12 岁儿童维生素 A 水平检测分析)
V35	Zhang YK et al	2007	Survey on Vit A deficiency in children under-6-years in Hebei Province (6 岁以下儿童不同年龄间维生素 A 缺乏情况对比研究)
V36	Ma JM et al	2007	Analysis of mass survey of Vit A level of children’s blood plasma in Tianjin Suburbs (天津市非城市儿童血浆维生素 A 水平普查分析)
V37	Shi YH et al	2010	Nutritional status and effect factors of infants of Mongolian and Han nationality aged 0-2 years old in a certain area of Inner Mongolia Automatic Region (内蒙古某地区 0-2 岁蒙、汉族婴幼儿营养状况及其影响因素)

V38	Wang Q et al	2005	Analysis of nutritional status of Vitamin A and its influence factors of the students in a boarding school* (某寄宿制小学学生维生素 A 营养状况及影响因素分析)
V39	Liu LG et al	1995	Survey of VA deficiency and nutritional status of preschool children in Wuhan City* (武汉市学龄前儿童 VA 缺乏及其营养状况调查)
V40	Zhang L et al	2010	Study of Vitamin A levels and its relevant factors in children aged under 5 in Jilin Province* (吉林省 5 岁以下儿童维生素 A 水平及相关因素研究)
V41	Xu BR et al	2004	Study on the status of subclinical vitamin A deficiency in children under 5 years of age (江苏省 5 岁以下儿童亚临床维生素 A 缺乏现状研究)
V42	Wei HM et al	2009	Survey of nutrition-related diseases in children aged seven to ten years old in Longgang District, Shenzhen (龙岗区 7-10 岁儿童营养相关疾病现状分析)
V43	Jin HJ et al.	2009	Level of serum Vitamin A in children under 5 years old and its influence factors in Liandu District, Lishui City (丽水市莲都区 5 岁以下儿童血清维生素 A 水平及影响因素分析)
V44	Wang XT et al.	2007	Survey of Vitamin A deficiency of children aged 1-6 in Heping District, Shenyang City* (沈阳市和平区 1~6 岁儿童维生素 A 缺乏调查)
V45	Wen H et al.	2008	Study of Vitamin A levels and its relevant factors in collective children aged 3-6 in Shenyang City* (沈阳市 3~6 岁集体儿童维生素 A 水平与相关因素调查)
V46	Lin L et al.	2008	Evaluation on growth, anaemia and vitamin A deficiency of rural children (贫困农村儿童生长发育、贫血及维生素 A 营养评价)
V47	Yang RW et al.	2008	Investigation on plasma vitamin A deficiency of children aged 0-4 years and risk factors in Zhejiang province (浙江省 0~4 岁儿童血清维生素 A 水平抽样调查及影响因素分析)
V48	He QF et al.	2006	Study and analysis on Vitamin A deficiency for children of school age from 7 to 12 years old in Zhejiang Province (浙江省 7～12 岁学龄儿童维生素 A 缺乏情况调查分析)
V49	Yang RL et al.	2000	Investigation the serum Vitamin A levels of children aged 0-6 in Zhejiang Province (浙江省 0～6 岁儿童血清维生素 A 水平调查)
V50	Qiu XG et al.	2008	Study of Vitamin A deficiency prevalence and its influence factors of children aged under5 in Fuzhou City* (福州市 5 岁以下儿童维生素 A 缺乏患病率及影响因素的流行病学调查)

V51	Lu XY et al	2001	A study on the Vitamin A levels and its influential factors in children under 5 years of age in Beijing (北京市 5 岁以下儿童维生素 A 水平及影响因素研究)
V52	Lin LM et al	2002	Survey on vitamin A deficiency in children under-6-years in China (中国六岁以下儿童维生素 A 缺乏情况调查)
V53	Hu XJ et al	2008	Analysis of children with subclinical VA deficiency (儿童维生素 A 亚临床缺乏分析)
V54	Li RL et al	2005	Levels of vitamin A, ferritin and bone calcium protein and nutritional status of infants in Qinba mountainous area (秦巴山区婴幼儿维生素 A、铁蛋白、骨钙素水平及营养状况调查)

*Note: The English titles were obtained from articles or translated by the investigators (marked with *)*

Table S3. Detailed characteristics of the included studies (n=54).

Study ID	Author	Year Published	Province	Setting	Gender	Sampling	Investigation Year	Age range (year)	Sample size	NO. of VAD cases	NO. of MVAD cases
V01	Liu YF et al.	2012	Chongqing	Rural	Both	Stratified cluster sampling	2008	3-6	381	5	79
V02	Yang C et al.	2016	National	Both	Both	Stratified multistage cluster sampling	2002	6-13	2002:8170; 2012:6016	2002:731; 2012:492	2002:3732; 2012:1312
V03	Zhang YW	2003	Anhui	Both	Mixed	Random stratified cluster sampling	2002	0-5	1052	72	477
V04	Xu JH et al.	2003	Liaoning	Urban	Mixed	Random cluster sampling	2001	2-6	2585	84	447
V05	Sun LF et al.	2016	Beijing	Both	Mixed	Random stratified cluster sampling	2014	2-6	487	189	236
V06	Chen Z et al.	2002	Hubei	Both	Both	Stratified cluster sampling	2000	0+	855	108	
V07	Qiu XG et al.	2010	Fujian	Mixed	Mixed	Stratified cluster sampling	2008	0-5	5973	190	1374
V08	You Y	2010	Gansu	Rural	Mixed	Stratified cluster sampling	2007	7-13	236	5	20
V09	Liu T et al.	2016	Gansu	Rural	Mixed	Random cluster sampling	2015	912	296	15	34
V10	Qi ZQ et al.	2001	Qinghai	Both	Mixed	Stratified cluster sampling	2000	0-5	609	109	283
V11	Fang ZF et al.	2007	Guangxi	Rural	Both	Random stratified cluster sampling	2002	3-12	316	79	139
V12	Fu SL et al.	2006	Anhui	Both	Both	Stratified cluster sampling	2004	0-5	1085	82	512

V13	Yang Q et al.	2002	Hubei	Both	Mixed	Random stratified cluster sampling	2000	0-6	624	29	220
V14	Lu L et al.	2001	Jiangsu	Urban	Mixed	Random cluster sampling	2000	0-4	428	58	
V15	Hu HF	2002	Shandong	Both	Mixed	Stratified sampling	2001	0-8	1800	206	648
V16	He YF et al.	2005	Jiangsu	Urban	Both	Stratified cluster sampling	2004	2-12	2529	24	470
V17	Cai LR et al.	2010	Fujian	Both	Mixed	Stratified cluster sampling	2009	0-5	5237	243	1688
V18	Huang HY et al.	2004	Fujian	Both	Mixed	Random cluster sampling	2002	0-5	3029	65	362
V19	Fan P et al.	2012	Shandong	Rural	Mixed	Cluster sampling	2009	0.5-2	254	103	83
V20	Zhou YX et al.	2002	Shandong	Both	Mixed	Random stratified cluster sampling	2000	0-6	613	48	202
V21	Zhao JJ et al.	2016	Shaanxi	Rural	Both	Random cluster sampling	2014	6-17	1073	41	635
V22	Zhang YF et al.	2002	Sichuan	Both	Mixed	Random stratified sampling	2000	0-5	617	34	194
V23	Zhou ZX	2001	Hubei	Both	Both	Random stratified sampling	2000	0-5	750	108	
V24	Yang SP et al.	2011	Hubei	Urban	Mixed	Random stratified cluster sampling	2008	0-5	1270	43	93
V25	Mi J et al.	2003	Tibet	Both	Mixed	Random stratified cluster sampling	2001	0-5	1257	106	483
V26	Zhong CM	2010	Shandong	Rural	Both	Cluster sampling	2008	0.58	278	107	65
V27	Jiang HP et al.	2012	Chongqing	Mixed	Both	Random cluster sampling	2009	10-19	356	52	181
V28	Wu ZY	2012	Henan	Both	Mixed	Stratified cluster sampling	2009	0-5	998	69	477

V29	Zhao ZY et al	1998	Zhejiang	Rural	Mixed	Random sampling	1994	0.5-7	2500	229	176
V30	Liu JW et al	2008	Hunan	Both	Both	Stratified cluster sampling	2002	3-12	520	54	239
V31	Wang HD et al	2001	Henan	Urban	Mixed	Random stratified sampling	1998	0-10	240	20	54
V32	Chen K et al	2008	Chongqing	Rural	Both	Stratified cluster sampling	2005	2-5	455	30	120
V33	Chen HL et al	2000	Zhejiang	Both	Mixed	Stratified cluster sampling	1998	0.5-7	843	85	
V34	Wang R et al	2010	Gansu	Both	Both	Stratified cluster sampling	2009	7-12	184	20	31
V35	Zhang YK et al	2007	Hebei	Mixed	Mixed	Random stratified sampling	2004	0-6	1473	63	558
V36	Ma JM et al	2007	Tianjin	Rural	Mixed	Cluster sampling	2004	0-8	47076	1130	8191
V37	Shi YH et al	2010	Inner Mongolia	Rural	Mixed	Cluster sampling	2004	0-2	261	113	
V38	Wang Q et al	2005	Henan	Urban	Mixed	Cluster sampling	2003	6-8	100	32	
V39	Liu LG et al	1995	Hubei	Urban	Mixed	Cluster sampling	1992	2-7	362	39	
V40	Zhang L et al	2010	Jilin	Both	Mixed	Random cluster sampling	2007	0-5	1220	136	345
V41	Xu BR et al	2004	Jiangsu	Both	Both	Random stratified sampling	2000	0-5	1170	154	
V42	Wei HM et al	2009	Guangdong	Urban	Both	Cluster sampling	2006	7-10	3836	228	596
V43	Jin HJ et al.	2009	Zhejiang	Urban	Mixed	Random cluster sampling	2005	0-5	158	2	11

V44	Wang XT et al.	2007	Liaoning	Urban	Mixed	Random cluster sampling	2006	1-6	6143	55	2323
V45	Wen H et al.	2008	Liaoning	Both	Both	Random stratified cluster sampling	2006	3-6	15519	676	3676
V46	Lin L et al.	2008	Sichuan	Rural	Both	Random cluster sampling	2005	3-10	409	33	98
V47	Yang RW et al.	2008	Zhejiang	Mixed	Mixed	Random stratified cluster sampling	2005	0-4.9	357	11	26
V48	He QF et al.	2006	Zhejiang	Both	Both	Random stratified cluster sampling	2002	7-12	1799	93	643
V49	Yang RL et al.	2000	Zhejiang	Mixed	Mixed	Random stratified cluster sampling	1998	0-6	1842	145	569
V50	Qiu XG et al.	2008	Fujian	Urban	Mixed	NS	2005	0-5	571	15	73
V51	Lu XY et al	2001	Beijing	Both	Mixed	Cluster sampling	2000	0-5	642	32	213
V52	Lin LM et al	2002	14 provinces	Both	Mixed	Strafitied sampling	2000	0-6	8669	1018	3396
V53	Hu XJ et al	2008	Hubei	Mixed	Both	Cluster sampling	2007	2-6	5409	111	433
V54	Li RL et al	2005	Shaanxi	Rural	Mixed	Stratified cluster sampling	2002	0-3	123	17	

Table S4. The deviance information criterion (DIC) difference relative to the intercept-only model*.

Variable	VAD	MVAD
	DIC difference	DIC difference
Age	-47.5	-28.0
Setting	-23.4	-16.9
Investigation year	4.6	30.4
Gender	7.3	0.2
Age and Setting	-59.5	-38.2

**DIC difference=DIC for model with variable(s) of interest - DIC for intercept-only model; The effect of gender was based on studies that reported the prevalence estimates for both boys and girls; The effect of investigation year was based on studies conducted after the year 2000.*
